# Lithium Carbonate as Treatment for Paliperidone Long-Acting Injection-Induced Leukopenia and Neutropenia in a Patient With Schizophrenia: A Case Report

**DOI:** 10.7759/cureus.116725

**Published:** 2026-09-22

**Authors:** Manar Alnabelsi, Lina Elsayed, May Arman, Ali Alkot

**Affiliations:** 1 Medicine and Surgery, HMS (Health and Medical Services) Mirdif Hospital, Dubai, ARE; 2 Psychiatry, Al Amal Psychiatric Hospital, Dubai, ARE

**Keywords:** antipsychotics, leukopenia, lithium carbonate, neutropenia, paliperidone, schizophrenia, second‑generation antipsychotics

## Abstract

Schizophrenia is a chronic psychiatric disorder characterized by disturbances in thought processes, perception, emotional responsiveness, and social functioning. Second‑generation antipsychotics, including clozapine, risperidone, olanzapine, and quetiapine, may rarely cause hematologic complications such as leukopenia and neutropenia. Paliperidone, the active metabolite of risperidone, has also been associated with these adverse effects. Leukopenia refers to reduced white blood cell (WBC) count, often due to neutropenia, defined as an absolute neutrophil count (ANC) of <1.50 × 10⁹/L. Lithium carbonate has been reported in case literature to increase neutrophil counts in antipsychotic‑associated neutropenia, although its use remains off‑label and is not included in formal treatment guidelines.

We report a 23‑year‑old male with schizophrenia who developed progressive leukopenia and neutropenia following treatment with long‑acting paliperidone. After initial stabilization on oral risperidone and monthly paliperidone injections, the patient was transitioned to a three‑monthly paliperidone formulation. Routine laboratory monitoring revealed a decline in ANC and WBC levels. Other medical causes were excluded, and drug‑induced neutropenia was suspected. Lithium carbonate 400 mg/day was initiated, resulting in partial improvement of hematologic parameters. However, fluctuations persisted due to intermittent non‑adherence, confirmed by a subtherapeutic lithium level. Adjusting paliperidone to a lower monthly dose and reinforcing lithium adherence led to stabilization of ANC and WBC counts. The patient remained psychiatrically stable throughout follow‑up.

## Introduction

Schizophrenia is a mental disorder marked by impairments in thought processes, perceptions, emotional responsiveness, and social interactions. Although its course varies among individuals, schizophrenia is typically persistent and can be severe and disabling. Symptoms include psychotic features, such as hallucinations, delusions, and thought disorder, as well as reduced emotional expression, diminished motivation, impaired social relationships, motor abnormalities, and cognitive deficits [1].

The dopamine hypothesis remains the leading model for explaining the mechanism of antipsychotic medications, which primarily target dopamine dysregulation [2]. While these medications are essential for managing schizophrenia, serious hematologic complications, including leukopenia and neutropenia, are recognized adverse effects. Clozapine is most frequently associated with these complications, but similar events have been reported with other second‑generation antipsychotics such as risperidone, olanzapine, and quetiapine. Paliperidone, the primary active metabolite of risperidone (9‑hydroxyrisperidone), has also been implicated in rare cases of leukopenia and neutropenia [3].

Leukopenia is defined as a reduction in total white blood cell (WBC) count, often secondary to neutropenia, which is characterized by an absolute neutrophil count (ANC) of less than 1.50 ×10⁹/L. Standard management of antipsychotic‑induced neutropenia primarily involves discontinuation or dose reduction of the offending agent, accompanied by close hematologic monitoring [4]. Hematologic adverse effects have also been described more broadly across atypical antipsychotics [5]. Lithium carbonate may be used as an off‑label adjunct to increase neutrophil production in situations where continuation of antipsychotic therapy is clinically necessary, although its use in paliperidone‑associated cases remains limited [6].

This case report describes a rare instance of paliperidone‑associated neutropenia in a male patient with schizophrenia, emphasizing the need for timely recognition and individualized management when hematologic abnormalities emerge during antipsychotic therapy.

## Case presentation

Patient A is a 23‑year‑old male who began presenting psychotic symptoms consistent with DSM‑IV‑TR (Fourth Edition, Diagnostic and Statistical Manual of Mental Disorders, Text Revision) diagnostic criteria for schizophrenia. His symptoms first appeared in 2019, including persecutory delusions, feeling watched, believing others intended to harm him, disorganized thinking, behavioral disturbances, and aggression toward his surroundings. He had no premorbid psychiatric symptoms, no family history of psychosis, and no other mental disorders. In 2020, he sought psychiatric care and was started on olanzapine 10 mg, which he continued for one year with significant improvement before discontinuing it independently, leading to recurrence and worsening of symptoms. In 2022, psychosocial stressors and financial difficulties further exacerbated his condition, resulting in increased agitation and aggression.

In January 2023, the patient was brought to the emergency department due to aggressive behavior and was admitted to the inpatient psychiatry unit at Al Amal Hospital. He was started on risperidone, titrated to 3 mg twice daily (BID), and received paliperidone palmitate 100 mg intramuscular (IM) monthly (Invega Sustenna®) after confirming a baseline absolute neutrophil count (ANC) of 3.13 × 10⁹/L. Following discharge, he remained clinically stable during outpatient follow‑up. Risperidone was gradually reduced over the next two months and discontinued in April 2023 to avoid antipsychotic polypharmacy, leaving paliperidone as the primary agent.

After 6 months of stability on monthly paliperidone, he was transitioned in October 2023 to paliperidone palmitate 350 mg IM every 3 months (Invega Trinza®). Six months after this switch, routine laboratory monitoring revealed a decline in ANC to 1.17 ×10⁹/L, consistent with mild neutropenia, and a WBC count of 3.24 ×10⁹/L. One month later, the ANC further decreased to 1.12 ×10⁹/L (mild neutropenia). Alternative causes of leukopenia and neutropenia were systematically evaluated, including complete metabolic panel, liver and renal function tests, thyroid profile, viral serologies (HIV, hepatitis B/C, Epstein-Barr virus (EBV), cytomegalovirus (CMV)), autoimmune screening (antinuclear antibody (ANA)), and nutritional assessment (vitamin B12 and folate). All results were within normal limits. The patient had no recent infections, no exposure to myelosuppressive medications, no substance use, and no chronic medical illness.

Given the temporal relationship with paliperidone dose escalation and the exclusion of other etiologies, drug‑induced neutropenia was suspected, and lithium carbonate 400 mg/day was initiated in May 2024. Discontinuation of paliperidone or switching to an alternative antipsychotic was initially avoided because the patient had achieved sustained psychiatric stability on paliperidone after multiple prior relapses and episodes of severe agitation. Given his history of non‑adherence and aggression, maintaining continuity with a long‑acting injectable formulation was prioritized to reduce the risk of psychiatric decompensation. At the time lithium was introduced, the neutropenia was in the mild‑to‑moderate range, allowing for a trial of adjunctive therapy rather than immediate antipsychotic discontinuation. A switch to a lower monthly paliperidone dose was later implemented when ANC values continued to decline.

According to the manufacturer’s prescribing information for paliperidone palmitate (Invega Sustenna® and Invega Trinza®), treatment discontinuation should be considered when the absolute neutrophil count (ANC) falls below 1000/mm³, with immediate discontinuation recommended for severe neutropenia (<500/mm³). In this case, the patient’s ANC decline initially remained within the mild‑to‑moderate range (1.12-1.17 ×10⁹/L), above the discontinuation threshold, allowing continuation of therapy with close monitoring. When the ANC later decreased to 0.73 ×10⁹/L, approaching the manufacturer’s threshold for discontinuation, the three‑monthly formulation was stopped and replaced with a lower‑dose monthly paliperidone injection. This adjustment aligned with product guidance while balancing the need to maintain psychiatric stability in a patient with a history of severe relapse and aggression.”

After starting lithium carbonate, hematologic parameters initially improved (ANC 1.83 ×10⁹/L; WBC 4.35 ×10⁹/L). However, one month later, the ANC again decreased to 1.66 ×10⁹/L (low‑normal range). Consequently, the three‑monthly paliperidone formulation was discontinued, and the patient was switched to a lower monthly dose of paliperidone palmitate 75 mg. This change reflected discontinuation of the three‑monthly paliperidone palmitate formulation rather than discontinuation of paliperidone therapy itself, as treatment was continued using the monthly long‑acting injectable formulation at a lower dose. Despite these adjustments, in September 2024, the ANC dropped to 0.73 ×10⁹/L, indicating moderate neutropenia approaching the severe threshold, representing the lowest value recorded during follow‑up. When the ANC reached its nadir of 0.73 ×10⁹/L, the patient was closely monitored, and the decline prompted reconsideration of the antipsychotic regimen. Hematology was not consulted at the time of the nadir, and no additional hematologic investigations such as peripheral smear or bone marrow studies were performed. Earlier in the course, a comprehensive evaluation-including viral serologies, autoimmune screening, nutritional assessment, and metabolic testing-had already been completed and was unremarkable, and subsequent management focused on close monitoring and adjustment of the antipsychotic regimen. The three‑monthly paliperidone palmitate formulation was discontinued, and treatment was continued using the lower‑dose monthly formulation. No infectious symptoms were reported at the time of the nadir, and repeat blood counts were obtained as part of routine follow‑up, demonstrating subsequent improvement.

A single subtherapeutic lithium concentration (0.17 mmol/L) indicated low lithium exposure at the time of measurement, but it does not definitively establish that non‑adherence caused the earlier ANC fluctuations. The lithium level was obtained during routine follow‑up approximately one month after initiation, and no additional serum levels were available. The patient reported missing doses during this period, which was consistent with the low serum concentration. After identifying the subtherapeutic level, adherence was reinforced through direct counseling and clarification of dosing instructions. Subsequent stabilization of ANC and WBC values occurred after these measures, with counts improving to 2.65 ×10⁹/L and 5.71 ×10⁹/L, respectively. Throughout the treatment course, the patient remained psychiatrically stable, with no recurrence of aggressive behavior or psychotic symptoms. A summary of the patient’s hematologic values and medication adjustments is provided in Table 1.

**Table 1:** Timeline of paliperidone dose adjustments, lithium initiation, and serial ANC/WBC values Summary of hematologic parameters, paliperidone dosing, and lithium initiation over the clinical course. Laboratory values include white blood cell count (WBC) and absolute neutrophil count (ANC), presented with corresponding medication adjustments.

Date	Paliperidone Dose/Formulation	Lithium Dose	ANC (×10⁹/L)	WBC (×10⁹/L)	Notes
Jan 2023	100 mg monthly (Invega Sustenna)	-	3.13	-	Baseline
Apr 2023	100 mg monthly	-	-	-	Risperidone discontinued
Oct 2023	350 mg q3mo (Invega Trinza)	-	-	-	Switched to LAI, First dose of 3‑monthly formulation
Apr 2024	350 mg q3mo	-	1.17	3.24	First notable decline
May 2024	350 mg q3m	400 mg/day	1.12	2.89	Lithium started
Jun 2024	350 mg q3mo	400 mg/day	1.83	4.35	Partial improvement
Jul 2024	75 mg monthly	400 mg/day	1.66	3.73	Three‑monthly LAI discontinued; monthly LAI continued at lower dose
Aug 2024	75 mg monthly	400 mg/day	1.71	4.57	Mild improvement
Sep 2024	75 mg monthly	400 mg/day	0.73	2.89	Severe drop (ANC nadir)
Oct 2024	75 mg monthly	400 mg/day	1.97	4.93	Improved
Nov 2024	75 mg monthly	400 mg/day	1.49	3.47	Non‑adherence
Dec 2024	75 mg monthly	400 mg/day	2.65	5.71	Stabilized

## Discussion

Antipsychotic medications are associated with a wide range of adverse effects, with hematologic toxicity, particularly leukopenia and neutropenia, being among the most serious. Clozapine is the antipsychotic most frequently linked to these complications and therefore requires routine hematologic monitoring [6]. Although the exact pathophysiological mechanisms underlying antipsychotic‑induced neutropenia remain unclear, proposed mechanisms include direct bone marrow suppression, antibody formation against hematologic precursors, and peripheral destruction of neutrophils [4].

The patient’s ANC values ranged from mild neutropenia (1.0-1.5 ×10⁹/L) to moderate neutropenia (0.5-1.0 ×10⁹/L), with one episode approaching the severe threshold. This classification is clinically relevant, as management intensity increases with severity. Mild neutropenia typically warrants close monitoring, whereas moderate neutropenia may require dose reduction or discontinuation of the offending agent. In this case, the patient’s moderate decline, combined with the temporal association with paliperidone dose escalation, justified both lithium initiation and subsequent adjustment of antipsychotic therapy.

Given the temporal association with paliperidone initiation and dose escalation, and the exclusion of other etiologies, drug‑induced neutropenia was considered the most likely diagnosis. A formal adverse drug reaction assessment using the Naranjo algorithm yielded a score of 6, indicating a probable relationship between paliperidone and the development of neutropenia [7]. This score reflected the temporal association with dose escalation, improvement after dose reduction, absence of alternative medical causes, and recurrence during continued exposure.

Taken together, the delayed but reproducible decline in ANC following paliperidone dose escalation, and the subsequent improvement after dose reduction, support a probable causal relationship between exposure to paliperidone and the development of neutropenia. Paliperidone therapy began in January 2023, the three‑monthly formulation was introduced in October 2023, and the first documented neutropenia occurred in April 2024. This relatively delayed onset, combined with continued exposure to paliperidone, reduces the certainty of a direct causal relationship, although delayed hematologic reactions to long‑acting antipsychotics have been reported. The decline in ANC closely followed the transition to the higher‑dose three‑monthly formulation, supporting a dose‑dependent effect. The delayed onset therefore reduces, but does not eliminate, the likelihood of causation and remains consistent with a probable adverse drug reaction based on the Naranjo assessment.

This case also demonstrates the potential therapeutic contribution of adjunctive lithium carbonate in managing paliperidone‑associated neutropenia. The patient developed progressive hematologic abnormalities after the initiation of paliperidone, which improved following dose reduction and the introduction of lithium carbonate. Neutropenia occurring after the initiation of antipsychotic therapy is considered a rare adverse effect according to surveillance data [8].

To date, only a few case reports have described neutropenia or leukopenia associated with paliperidone. One report involving an African American patient documented the development of neutropenia shortly after starting paliperidone, which resolved within 16 days of discontinuation and switching to haloperidol [9]. Another case by Torres AR et al. described paliperidone‑induced neutropenia that improved after adjunctive treatment with lithium carbonate [8]. These findings align with our observation that lithium may offer a possible benefit in managing antipsychotic‑related hematologic abnormalities. Collectively, these reports support the two key elements observed in our patient: paliperidone has been implicated in the development of neutropenia, and lithium has demonstrated the ability to increase neutrophil counts in cases of antipsychotic‑related hematologic toxicity. Although the prior cases involved different antipsychotic agents or management strategies, the combined evidence provides a coherent framework that aligns with the clinical improvement seen following lithium initiation in our patient. However, the improvement in ANC cannot be attributed to lithium alone, as the paliperidone regimen was also modified during the same period. This concurrent medication change represents a confounding factor that limits the ability to draw causal conclusions regarding lithium’s effect on hematologic recovery.

In this patient, lithium carbonate appeared to support recovery of the absolute neutrophil count (ANC). Severe leukopenia and neutropenia significantly increase morbidity and mortality due to heightened susceptibility to infections and related complications [2]. This case therefore suggests that lithium may represent an adjunctive option when antipsychotic‑induced neutropenia is suspected, provided that appropriate monitoring is ensured.

## Conclusions

A 23‑year‑old male patient with schizophrenia developed paliperidone‑associated leukopenia and neutropenia. Hematologic parameters improved following paliperidone dose reduction and the introduction of lithium carbonate, although concurrent medication changes and fluctuations in ANC values prevent the attribution of efficacy to lithium alone. This case underscores the importance of careful hematologic monitoring when using antipsychotics, including paliperidone. Lithium may represent an adjunctive option when continuation of antipsychotic therapy is clinically necessary, provided that appropriate monitoring of serum lithium levels, renal and thyroid function, calcium, and relevant clinical parameters is ensured. In keeping with the single‑case nature of this report, lithium is described as a possible contributing factor rather than a demonstrated therapeutic intervention, and further study is required before any conclusions can be drawn regarding its hematologic effects. Lithium’s potential effect on neutrophil recovery is thought to occur through stimulation of granulopoiesis and increased release of neutrophils from the bone marrow, although evidence remains limited to case reports and small observational studies.
